# Virulence, Phylogenetic Grouping, and Antimicrobial Resistance Traits of Extraintestinal *Escherichia coli* in Clinical Isolates From Northwest Mexico

**DOI:** 10.1155/ijm/8881117

**Published:** 2025-09-19

**Authors:** Ana María Castañeda-Meléndrez, Patricia Catalina García-Cervantes, María Elena Báez-Flores, Aldo Francisco Clemente-Soto, Edwin Barrios-Villa, Rodolfo Bernal-Reynaga

**Affiliations:** ^1^Unit of Public Health Research “Dr. Kaethe Willms,” Doctorate in Biomedical Sciences, Faculty of Chemical-Biological Sciences, Autonomous University of Sinaloa, University City, Culiacan, Sinaloa, Mexico; ^2^Laboratory of Molecular Biology and Genomics, Department of Agricultural and Chemical-Biological Sciences, University of Sonora, Campus Caborca, Caborca, Sonora, Mexico

**Keywords:** antimicrobial resistance, extraintestinal pathogenic *Escherichia coli*, multidrug-resistant strains, phylogeny, virulence factors

## Abstract

*Escherichia coli* is a highly diverse bacterial species that is canonically commensal; however, its genomic characteristics have enabled its evolution into pathogens capable of causing extraintestinal infections (ExPEC), which pose significant clinical challenges because of the variety of sites that can infect (mainly the urinary tract) and the multidrug resistance associated with these strains. The present study is aimed at characterizing ExPEC isolates recovered from the wards of a hospital in Sinaloa, Mexico, to establish their virulence and antimicrobial resistance profiles, as well as the phylogenetic group. Then, 200 *Escherichia coli* isolates and their antimicrobial susceptibility were confirmed by the VITEK-2 automated system. Virulence factor genes and phylogenetic grouping were performed through endpoint and multiplex PCRs. Then, 59% of the strains produced extended-spectrum *β*-lactamases (ESBLs), and 71.5% were classified as multidrug resistant (MDR), exhibiting high resistance rates to antibiotics such as amoxicillin, ampicillin, ciprofloxacin, and various cephalosporins. In this sense, 68.5% and 33% of the isolates were positive for the *blaCTX-M1-8* and *blaCTX-M9* genes, respectively, both associated with resistance to cefotaxime. Furthermore, 37% of the isolates harbored the *blaOXA48* gene, which is linked to resistance to oxacillin-type *β*-lactams. Moreover, 143 (71.5%) of them were classified as MDR. Regarding virulence, the distribution of toxin genes such as *hlyA* and *vat* was 16.5% and 24.5%, respectively. Adhesins *papC* and *fimA* were found in 62% and 34%, respectively. Additionally, the iron acquisition systems and outer membrane proteins such as *iutA* (74%), *fyuA* (63%), *iroN* (10%), *agn43* (82%), and *kpsmTII* (34.5%) were present in the isolates. Phylogenetic analysis showed a predominance of group B2 (46%), followed by groups A (13.5%) and E (10.5%). These findings highlight the complexity and challenges posed by ExPEC strains in terms of antimicrobial resistance and virulence relevant to public health in hospital settings.

## 1. Introduction

*Escherichia coli* inhabits the human intestine and plays a crucial role in the digestion and balance of the intestinal microbiota. However, a few strains of *E. coli* have evolved into potential pathogens that can cause various diseases in humans. These pathogenic strains pose a constant challenge to public health worldwide, as they can lead to diseases ranging from mild gastroenteritis to severe infections, such as sepsis and hemolytic uremic syndrome [1, 2]. Pathogenic *E. coli* strains are divided into two main groups: diarrheagenic *Escherichia* coli (DEC) and extraintestinal pathogenic *Escherichia coli* (ExPEC); these latter strains are specialized subsets of *E. coli* distinguished by their ability to cause infections in various parts of the body, outside of the gastrointestinal tract [3].

Among the most prevalent infections attributed to ExPEC are urinary tract infections (UTIs), associated with strains of uropathogenic *Escherichia coli* (UPEC). These pathogenic strains can ascend from the urethra to the bladder (where they cause cystitis) and the kidneys (pyelonephritis), resulting in distressing symptoms and potentially severe conditions [4]. Furthermore, ExPEC strains can potentially induce sepsis, a systemic inflammatory response to infection that poses a life-threatening risk. Sepsis generally originates from an infection source elsewhere in the body, such as the urinary tract or soft tissue, predominantly instigated by sepsis-associated *Escherichia coli* (SEPEC). Additionally, under certain circumstances, ExPEC can trigger infections in wounds, abscesses, or soft tissues, resulting in localized inflammation and infection. In some cases, particularly in neonates, ExPEC can be accountable for severe infections like sepsis and meningitis, which can have devastating consequences, primarily attributed to neonatal *Escherichia coli* meningitis (NEMEC) [5, 6].

The pathogenicity of these strains is intrinsically associated with a repertoire of virulence factors that facilitate their adherence to host cells, colonization of human tissues, evasion of the immune system, infliction of damage, and acquisition of essential nutrients required for growth and survival [7–9].

These *E. coli* strains can be classified into eight phylogroups, seven belonging to *E. coli sensu stricto* (A, B1, B2, C, D, E, F, and G) and one belonging to the *Escherichia* cryptic clades I–V [10–12]. Most strains responsible for extraintestinal infections belong to group B2, with some in group D, whereas commensal strains belong to groups A, B1, and C [13–15]. On the other hand, antibiotic resistance poses a mounting concern in the treatment of infections caused by pathogenic *E. coli* strains. The ability of these bacteria to acquire and disseminate antibiotic-resistant genes represents a substantial challenge in clinical therapy. The emergence of multidrug-resistant (MDR) ExPEC strains further complicates the management of these infections, rendering the selection of effective therapies a complicated task [16, 17].

In Mexico and particularly in Sinaloa, studies on ExPEC are scarce. One study reported the presence of MDR UPEC strains isolated from UTIs in pregnant women. The isolates had virulence factors, such as membrane-associated (*agn43*) and adhesins (*fimH* and *papC*), and resistance to multiple antibiotics was observed [18]. Other studies have identified strains belonging to the B2 phylogenetic group in clinical *E. coli* isolates from children with diarrhea and associated these characteristics with their antimicrobial resistance [19, 20].

Given the limited information available on ExPEC strains in northwestern Mexico and their potential clinical impact, this study is aimed at characterizing their antimicrobial resistance profiles, assessing the presence and distribution of key virulence factor genes, and determining their phylogenetic classification. We hypothesized that these clinical isolates would exhibit high levels of multidrug resistance and harbor a diverse repertoire of virulence genes, reflecting their adaptation to extraintestinal pathogenicity and their potential threat to public health in hospital settings.

In this sense, the present study is aimed at molecularly characterizing extraintestinal *E. coli* strains derived from diverse clinical isolates and establishing their virulence and antimicrobial resistance profile and phylogenetic group.

## 2. Materials and Methods

### 2.1. Clinical Isolates

Clinical isolates were obtained from inpatients and outpatients at the General Hospital of Culiacan in the period from July 2022 to March 2023; samples were obtained from different anatomical sites. Isolates identified as *E. coli* through Vitek 2 (BioMérieux, France) were transported to the Public Health Research Unit “Dr. Kaethe Willms” of the Faculty of Chemical and Biological Sciences at the Autonomous University of Sinaloa for subculturing on MacConkey agar plates from 18 to 24 h at 37°C.

### 2.2. Antimicrobial Susceptibility Profile of the Isolates

Antibiotic susceptibility testing was performed using AST-N402 cards in the Vitek 2 automated system (BioMérieux, France) according to CLSI 2024 specifications [21]. VITEK 2 automatically categorizes isolates as susceptible, intermediate, or resistant based on CLSI-defined minimum inhibitory concentration (MIC) breakpoints for the tested antibiotics in the automated antimicrobial susceptibility test (Table S1). The antibiotics considered included aminopenicillins (ampicillin), *β*-lactam combination agents (ampicillin–sulbactam and piperacillin–tazobactam), first-generation cephalosporins (cephalothin), second-generation cephalosporins (cefuroxime), third-generation cephalosporins (cefotaxime, ceftazidime, and ceftriaxone), fourth-generation cephalosporins (cefepime), penicillins (piperacillin), cephems (cefepime and ceftriaxone), aminoglycosides (gentamicin and amikacin), fluoroquinolones (ciprofloxacin), nitrofurans (nitrofurantoin), carbapenems (meropenem and ertapenem), and folate-pathway antagonists (trimethoprim–sulfamethoxazole).

### 2.3. Genomic DNA Extraction

DNA was extracted using the heat-shock method. Briefly, five bacterial colonies were resuspended in 1-mL nuclease-free water. The suspension was homogenized with a vortex and immersed in water at 100°C for 1 min. The bacterial lysate was transferred to ice for 20 min and stored at −20°C until use.

### 2.4. Identification of Virulence Factor Genes

The *hlyA* gene (1177 bp) (Table 1) was identified using conventional endpoint PCR. The reaction mixture included 2X Green GoTaq Flexi Buffer (Promega), 2.5 U of GoTaq Flexi DNA Polymerase (Promega), 2 mM MgCl_2_ (Promega), 250 *μ*M of the dNTP mix (Promega), 0.6 *μ*M of each oligonucleotide, and 50 ng of DNA for a final volume of 25 *μ*L. The amplification protocol consisted of an initial denaturation cycle at 95°C for 5 min, followed by 35 cycles of 94°C for 30 s, 66°C for 40 s, and 68°C for 1 min, and a final extension cycle at 72°C for 10 min. The strain O30U was used as the positive control [29].

The *vat*, *papC*, *fimA*, and *fyuA* genes were amplified by multiplex PCR, referred to as ExPEC. The reaction mixture included 2X Green GoTaq Flexi Buffer (Promega), 1.5 U of GoTaq Flexi DNA Polymerase (Promega), 1.6 mM MgCl_2_ (Promega), 100 *μ*M dNTPs (Promega), the mixture of the four pairs of oligonucleotides (Table 1), and 50 ng of DNA, for a final volume of 25 *μ*L. The amplification protocol involved an initial denaturation cycle at 95°C for 5 min, followed by 35 cycles of 95°C for 5 min, 94°C for 30 s, 61°C for 1 min, and a final extension cycle at 72°C for 10 min. Clinical isolates 2H25B, V27, and O30U were used as positive controls [28].

Additionally, *iutA*, *agn43*, *kpsmTII*, and *iroN* were identified by multiplex PCR referred to as UPEC. The reaction mixture consisted of 1X Green GoTaq Flexi Buffer (Promega), 0.5 U of GoTaq Flexi DNA Polymerase (Promega), 1.8 mM of MgCl_2_ (Promega), 62 *μ*M of the dNTP mix (Promega), the mix containing the four pairs of oligonucleotides, and 50 ng of DNA, for a final volume of 25 *μ*L. The amplification protocol included an initial denaturation step at 95°C for 5 min, followed by 35 cycles of denaturation at 94°C for 30 s, annealing at 63°C for 30 s, extension at 68°C for 1 min, and a final extension step at 72°C for 10 min, using the V27 strain as a positive control.

### 2.5. Determination of Phylogenetic Group

All isolates were subject to phylogenetic analysis according to Clermont et al. [11] and classified as belonging to one of the eight phylogenetic groups (A, B1, B2, C, D, E, and F). The reaction mixture for this protocol included 1X Green GoTaq Flexi Buffer (Promega), 1.5 U of GoTaq Flexi DNA Polymerase (Promega), 1.6 mM MgCl_2_ (Promega), 100 *μ*M dNTPs (Promega), the mixture of the oligonucleotides (Table 2), and 50 ng of DNA, for a final volume of 10 *μ*L.

PCR reactions were performed under the following conditions: denaturation for 4 min at 94°C, 30 cycles of 5 s at 94°C and 20 s at 57°C (group E) or 59°C (quadruplex and group C), and a final extension step of 5 min at 72°C. The primers used for the allele-specific phylo-groups E and C PCRs were ArpAgpE.f and ArpAgpE.r and trpAgpC.f and trpAgpC.r, respectively, using the strains CFT073 and ATCC 25922 as positive controls [11, 31].

### 2.6. Identification of *β*-Lactamase Genes

All isolates were subjected to the detection of genes associated with *β*-lactamase production by endpoint PCR. Each reaction contained 0.4 *μ*M of each primer, GoTaq MasterMix (Promega Corporation, Madison WI, United States) (including reaction buffer, 400 *μ*M of each dNTP, 3 mM MgCl_2_, and 1× Taq DNA polymerase) to a final volume of 10 *μ*L. PCR reactions were performed under the following conditions: denaturation for 1 min at 94°C, 35 cycles of 1 min at 94°C, 40 s at 55°C, 1 min at 72°C, and a final extension step of 10 min at 72°C. The primers used are in Table 3, using the strain AL74 as the positive control.

All PCR products were obtained using a MiniAmp Plus thermal cycler (Applied BioSystems, United States). The amplicons were resolved on a 1.5% agarose gel electrophoresis and stained with Ultra GelRed (10,000×) (Bio-Sigma) and visualized using a UVP Transilluminator (AnalytikJena, United States).

Quality control was ensured by including positive control strains for each PCR assay. Clinical isolates previously characterized and confirmed to carry the relevant virulence genes such as O30U, V27, and 2H25B were used as positive controls in the amplification of specific virulence factors. For phylogenetic group assignment, the reference strains *E. coli* CFT073 and ATCC 25922 were included as controls for specific allele amplification, following standard procedures. For the identification of genes associated with *β*-lactamase production, the control strain AL74 of *Leclercia adecarboxylata*, previously isolated from raw vegetables, was employed as a positive control. These controls allowed verification of PCR reagent performance, primer specificity, and amplification conditions throughout the study.

### 2.7. Visualization of Resistance Relationships

Resistance relationships were depicted using the online Circos Plot tool 23. The visualization was created based on binary data indicating whether each isolate was resistant or not to the antibiotics tested. Connections between antibiotics were determined by visually identifying shared resistance patterns among isolates, without performing any formal statistical analysis to measure the strength or significance of these links. Thus, the Circos plot serves as an exploratory visualization to suggest possible patterns of cross-resistance.

### 2.8. Statistical Analysis

The data were analyzed using GraphPad Prism Version 9 software. The association between phylogenetic groups and the presence of virulence genes was assessed using the Chi-square test and Fisher's test, as applicable. The level of significance was set at *p* < 0.05.

## 3. Results

### 3.1. Patients and Isolate Origin

A total of 200 *E. coli* isolates were collected at the General Hospital of Culiacan from July 2022 to March 2023. During this period, female patients (105 samples, 52.5%) were more affected by these infections than male patients (95 samples, 47.5%), with ages ranging from 9 to 93 years old. The isolates were obtained from different anatomical sites of the patients in the different hospital wards, including urine cultures (163), wounds (18), sputum (6), blood cultures (4), catheter tips (3), peritoneal fluid (2), hemodialysis (1), bronchial aspirates (1), otic (1), and ascitic fluid (1) (Table 4).

### 3.2. Antimicrobial Resistance of Isolates

**Figure 1:**
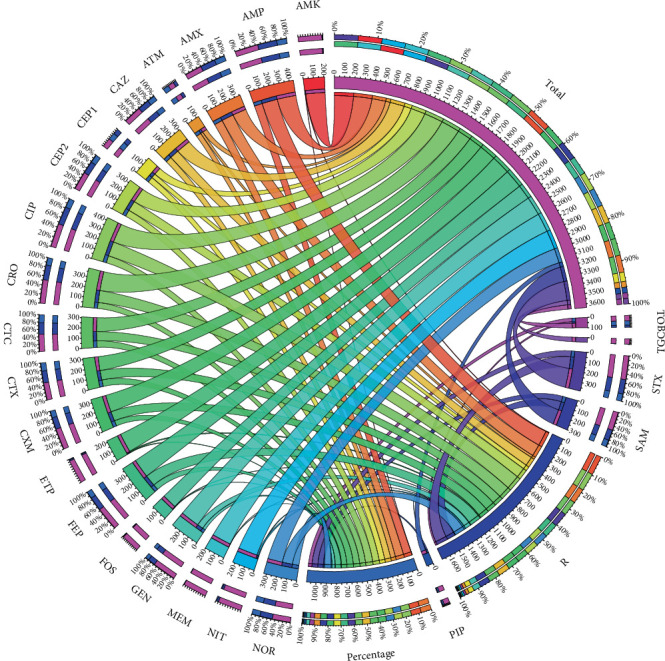
Relationships between different antibiotics by Circos plot. Antibiotics are displayed around the outer circle (ampicillin (AMP), ampicillin/sulbactam (SAM), piperacillin/tazobactam (PIP), cefazolin (CFZ1), cefazolin (urine) (CFZ2), ceftriaxone (CRO), cefepime (FEP), aztreonam (ATM), ertapenem (ETP), meropenem (MEM), amikacin (AMK), gentamicin (GEN), tobramycin (TOB), ciprofloxacin (CIP), tigecycline (TGC), nitrofurantoin (NIT), trimethoprim/sulfamethoxazole (STX), cephalothin (CEP), cefuroxime (CXM), cefuroxime axetil (CTX), cefotaxime (CTC), ceftazidime (CAZ), norfloxacin (NOR), fosfomycin (FOS), and amoxicillin (AMX)). The small bar charts above each label represent the percentage of isolates resistant to each antibiotic. Lines connecting antibiotics indicate shared resistance patterns across isolates and were inferred based on visual inspection of resistance data, without formal statistical testing. Line thickness corresponds to the number of shared resistant isolates between antibiotic pairs. This visualization provides a qualitative overview of potential cross-resistance relationships among antibiotic classes.

The antimicrobial resistance and the number of isolates assessed for each antibiotic are shown in Figure 1. High frequencies of resistance were observed against amoxicillin (87.5%), ampicillin (82.6%), ciprofloxacin (75.3%), cephalotin (69.2%), norfloxacin (68.6%), cefotaxime (67.3%), and cefuroxime–axetil (66.7%). Additionally, 59% of the isolates tested positive for extended-spectrum *β*-lactamases (ESBLs), as determined using AST-N402 cards in the Vitek 2 automated system (BioMérieux, France) according to CLSI 2024 specifications [21].

Additionally, 68.5% and 33% of the isolates tested positive for the *blaCTX-M1-8* and *blaCTX-M9* genes, respectively, both associated with resistance to cefotaxime. Furthermore, 37% of the isolates harbored the *blaOXA48* gene, which is linked to resistance to oxacillin-type *β*-lactams. Moreover, 143 (71.5%) of them were classified as MDR. The Circos plot (Figure 1) visually revealed several notable cross-resistance patterns. The most prominent associations involved resistance overlaps between ampicillin and amoxicillin, ciprofloxacin and norfloxacin, and among third- and fourth-generation cephalosporins such as cefotaxime, ceftriaxone, and cefepime. These findings suggest the presence of MDR phenotypes encompassing *β*-lactams and fluoroquinolones, which are commonly utilized in clinical practice.

### 3.3. Virulence Factor Genes and Phylogenetic Grouping of the Isolates

Regarding virulence genes in the isolates, the most prevalent gene found in the isolates was the one coding for *fimH* (95.5%), followed by *agn43* (82%), *fyuA* (63%), and *papC* (62%). Phylogenetic grouping showed that phylogroup B2 (46%) was the most prevalent among the isolates, then the phylogroups A (13.5%) and E (10.5%) (Table 5).

### 3.4. Most Represented Profiles of the Isolates With Multiple Virulence Genes

Based on genotyping, representative virulence profiles were identified, showing the combination of genes present in our isolates (Table 6). Interestingly, all isolates showed at least one of the virulence genes analyzed, and the combination of six genes (*papC + fyuA + kpsmTII + agn43 + iutA + fimH*) was the most prevalent in the isolates (17), and only two strains presented all the genes.

Contingency tables were constructed to assess the possible association between phylogenetic groups and the presence of virulence genes in the strains; however, no significant associations were observed, considering *p* values ≤ 0.05 as statistically significant.

## 4. Discussion

Pathogenic strains of *E. coli*, particularly ExPEC, are significant contributors to various infections and represent an ongoing public health concern [2]. The distribution of the isolates among patients, clinical characteristics, and hospital services provides valuable insight into the epidemiology of infections caused by these microorganisms.

In the present study, 200 ExPEC were evaluated to determine their antimicrobial resistance profile, identify virulence genes, and assign phylogenetic groups using PCR. The results highlight the diverse nature of infections in a hospital setting. Most isolates were associated with UTIs, reflecting the prevalence of this type of infection in the current clinical context. This finding is consistent with recent literature indicating that *E. coli* is the most common pathogen associated with UTIs, accounting for approximately 80%–90% of cases in both inpatient and outpatient settings [4, 33, 34]. Sex distribution showed an equitable representation in most categories, with a slight female predominance in the urine culture group. This observation can be attributed to the anatomical differences in women. Additionally, the ability of *E. coli* to colonize various anatomical sites is influenced by genomic plasticity and remodeling, which involves the acquisition or loss of genetic material. This process leads to the development of resistance or virulence factors. The severity of ExPEC infections can vary, influenced by specific virulence factors, host susceptibility, and immune response [35–38].

We observed a prevalence of MDR and ESBL production in 71.5% and 59% of isolates, respectively. This finding aligns with studies identifying the misuse of antibiotics as a critical factor in the development of resistant strains. Additionally, high resistance rates to common antibiotics amoxicillin (87.5%), ampicillin (82.6%), ciprofloxacin (75.3%), cephalotin (69.2%), norfloxacin (68.6%), cefotaxime (67.3%), and cefuroxime–axetil (66.7%) demonstrate the increasing resistance of *E. coli* to these drug classes.

The high resistance rates observed in our isolates, particularly to aminopenicillins, fluoroquinolones, and cephalosporins, can be understood within the broader context of antimicrobial use in Mexico. Studies have reported widespread empirical use of *β*-lactams and fluoroquinolones in both community and hospital settings, often without culture-based guidance, contributing to selective pressure and the emergence of MDR strains [17, 35]. Although national treatment guidelines (e.g., NOM-045-SSA2-2005 for UTIs) provide recommendations, adherence can vary across institutions. In addition, over-the-counter antibiotic access has historically contributed to misuse, although recent regulatory efforts have sought to reduce this practice.

At the molecular level, resistance in *E. coli* is commonly mediated by the production of ESBLs, particularly enzymes encoded by *blaCTX-M*, *blaTEM*, and *blaSHV*. In our study, 68.5% and 33% of the isolates tested positive for the *blaCTX-M1-8* and *blaCTX-M9* genes, respectively, both associated with resistance to cefotaxime. Furthermore, 37% of the isolates harbored the *blaOXA48* gene, which is linked to resistance to oxacillin-type *β*-lactams. These findings are consistent with recent global studies that report widespread dissemination of *blaCTX-M* variants, particularly *CTX-M-15*, in clinical settings. For example, a 2023 meta-analysis estimated a pooled prevalence of 37.55% for *blaCTX-M* among human *E. coli* isolates, while *blaOXA* was present in 16% of cases [39]. In a 2024 study from Tunisia, 92% of ESBL-producing *E. coli* carried *blaCTX-M* [40]. Notably, colocalization of *blaCTX-M* and *blaOXA-1* on IncI2-type plasmids has also been reported in China, suggesting a growing trend of mobile genetic elements facilitating the spread of multidrug resistance [41].

Several authors have reported that some strains may exhibit cross-resistance, defined as resistance to multiple distinct antimicrobial agents conferred by a single molecular mechanism [17, 42]. This trait may be present in our strains, as the Circos plot analysis suggests that strains developing resistance to one antibiotic often exhibit resistance to others within the same or different classes.

ExPEC strains harbored key virulence factors associated with pathogenicity. Among adhesins, *fimA*, *papC*, and *fimH* were prevalent in 34%, 62%, and 95.5% of isolates, respectively. These genes are essential for colonization and adhesion to host tissues, facilitating the development of persistent infections [43]. A study conducted in Mexico by Ballesteros-Monrreal et al. [26] evaluated 95 isolates from urinary tract infections and reported that *fimH* was the most prevalent virulence factor (100%), which aligns with the findings of this study (95.5%). The widespread presence of this adhesin, with reported prevalence ranging from 86% to 100% among clinically isolated UPEC strains, underscores its crucial role in the pathogenesis of urinary tract infections. Accordingly, *fimH* is widely recognized as a key molecular marker of virulence in UPEC, as documented by several authors worldwide [44–47].

Additionally, the prevalence of iron acquisition systems such as *iutA*, *fyuA*, and *iroN* (74%, 63%, and 10%, respectively) underscores the importance of efficient iron uptake for ExPEC survival and proliferation within the host [34, 47]. Regarding toxins, the genes *vat* (24.5%) and *hlyA* (16.5%) were identified. These virulence factors are thought to contribute to tissue damage and the severity of infections [7, 8].

Collectively, virulence factors contribute to ExPEC's ability to adhere to host cells, evade the immune system, and establish persistent infections. In this study, we identified multiple gene combinations, with the most prevalent profile—detected in 17 isolates being—*papC + fyuA + kpsmTII + agn43 + iutA + fimH*. This profile comprises three of the four virulence factors investigated, which are associated with adhesin production, membrane stability, and nutrient acquisition. Additionally, two isolates were found to harbor all the virulence factors analyzed in this study. This finding is particularly relevant, as the combination of genes from various functional groups supports previous discussions by several authors regarding the potential relationship between the number of virulence genes in ExPEC strains and their high pathogenicity, as well as their capacity to cause infections in various human anatomical sites [4, 9, 48, 49].

Another noteworthy finding is the identification of various hemolysis patterns among *hlyA*-positive isolates. Hemolysis is a complex phenomenon influenced by multiple factors, including the type and quantity of hemolysin produced, as well as the specific host environment [7]. Alpha hemolysis, observed in one-fourth of *hlyA*-positive isolates (data not shown), suggests diversity in the hemolytic activity of ExPEC strains.

The recent study by Praetorius in 2021 [50] expands the understanding of the cellular mechanisms affected by HlyA, demonstrating that this toxin not only causes physical damage through pore formation but also activates intracellular signaling pathways linked to inflammatory responses and programed cell death. These complex interactions enable ExPEC strains to persist within the host and exacerbate the severity of infections. Further research on factors affecting hemolysis patterns, such as variations in hemolysin expression or the presence of other regulatory elements, could provide valuable insights into the pathogenicity of these strains.

ExPEC isolates were assigned to eight major phylogenetic groups (A, B1, B2, C, D, E, F, and G). The predominant group was B2 (46%), followed by A (13.5%), E (10.5%), B1 (8%), D (8.5%), unknown (5%), F (4.5%), C (4%), and G (0%). This distribution slightly differs from the findings of Ballesteros-Monrreal et al. [14], who reported that ExPEC strains predominantly belong to phylogenetic groups B2 and D. These groups possess a higher number of virulence determinants compared to commensal strains, which belong to phylogenetic groups A and B1. Some reports suggest that UPEC can be distributed across all phylogenetic groups, with the migration of fecal strains to the urinary tract [13, 51].

A study conducted in Mexico by Bravata-Alcántara et al. [15] revealed that, of 107 isolates evaluated, most ExPEC isolates associated with extraintestinal infections belonged to phylogenetic group B2 (42.5%), followed by group A (27.1%), D (24.29%), and B1 (6.54%). The high prevalence of nonpathogenic strains from phylogenetic group A in this study may also be attributed to the migration of these intestinal strains to the urinary tract, where they serve as reservoirs of virulence factor genes and may become virulent through adaptations to pathogenic environments. Moreover, it is unclear whether *E. coli* strains should be classified as commensal or pathogenic based solely on the sample source or phylogenetic group, as phylogenetic group A can cause extraintestinal infections in immunocompromised individuals [51].

The predominance of phylogenetic group B2 (46%) in our isolates aligns with previous studies that describe this group as the most common among ExPEC strains worldwide. Group B2 is known to harbor a higher number of virulence-associated genes, including adhesins, iron acquisition systems, and toxins, which contribute to its enhanced pathogenic potential and adaptability in extraintestinal sites [2, 13, 48]. This group's genetic background may facilitate colonization, immune evasion, and persistence in clinical settings, particularly in urinary tract infections and sepsis.

Interestingly, a notable proportion of isolates belonged to phylogenetic group A (13.5%), which is generally associated with commensal *E. coli.* However, recent studies suggest that group A strains can acquire virulence and resistance determinants through horizontal gene transfer and may act as opportunistic pathogens, especially in immunocompromised hosts or under hospital selective pressures [14, 51]. The elevated presence of group A strains in this study may reflect such adaptive processes, highlighting the fluid boundaries between commensalism and pathogenicity within the *E. coli* species complex.

Understanding the molecular and phenotypic characteristics of ExPEC strains is essential for designing effective control and treatment strategies. The prevalence of antimicrobial resistance emphasizes the importance of prudent antibiotic use and the development of alternative therapeutic approaches. Moreover, identifying specific virulence factors and hemolysis patterns can aid in risk assessment and the prediction of clinical outcomes of ExPEC infections.

## 5. Conclusion

This investigation underscores the clinical and epidemiological significance of ExPEC strains in northwest Mexico (Sinaloa). Overall, the diversity in patient attributes, services, and sources of isolates highlights the versatility of *E. coli* in causing infections across different anatomical sites and patient populations. The data presented here contribute to a comprehensive understanding of the clinical and epidemiological aspects of ExPEC infections in the studied hospital setting. Further investigations could explore associations between specific patient characteristics and the severity or outcomes of ExPEC infections.

### 5.1. Limitations and Future Directions

This study has certain limitations that should be acknowledged. First, the lack of whole-genome sequencing (WGS) and multilocus sequence typing (MLST) restricted our ability to explore the clonal structure and potential transmission routes of the ExPEC isolates in depth. Although incorporating these techniques was beyond the scope of the present study, we recognize that their absence limits the genomic resolution of our findings. Despite this, we believe that epidemiological surveillance of potential pathogenic strains should be conducted using the available tools.

## Figures and Tables

**Table 1:** Primer sequences and amplicon sizes of the virulence genes.

**Protocol**	**Gene**	**Sequence (5**⁣′**-3**⁣′**)**	**Size (pb)**	**Concentration (pMol)**	**Reference**
Hemolysin	*hlyA*	F: AACAAGGATAAGCACTGTTCT	1177	1.5	[22]
		R: ACCATATAAGCGGTCATTCCC			

ExPEC	*papC*	F: ACGGCTGTACTGCAGGGTGTGGCG	328	1.5	[22]
		R: ATATCCTTTCTGCAGGGATGCAATA			
	*cnf1*	F: AAGATGGAGTTTCCTATGCAGGAG	498	1.5	[22]
		R: CATTCAGAGTCCTGCCCTCATTATT			
	*fimA*	F: GTTGTTCTGTCGGCTCTGTC	447	10	[22]
		R: ATGGTGTTGGTTCCGTTATTC			
	*fyuA*	F: TGATTAACCCCGCGACGGGAA	785	1.5	[23]
		R: CGCAGTAGGCACGATGTTGTA			
	*vat*	F: AGAGACGAGACTGTATTTGC	289	5	[24]
		R: GTCAGGTCAGTAACGAGCAC			

UPEC	*iroN*	F: AAGTCAAAGCAGGGGTTGCCCG	667	1.7	[24]
		R: GACGCCGACATTAAGACGCAG			
	*kpsmTII*	F: GCGCATTTGCTGATACTGTTG	578	30	[25]
		R: AGGTAGTTCAGACTCACACCT			
	*agn43*	F: CTGGAAACCGGTCTGCCCTT	433	2	[26]
		R: CCTGAACGCCCAGGGTGATA			
	*iutA*	F: GGCTGGACATCATGGGAACTGG	302	2.5	[23]
		R: CGTCGGGAACGGGTAGAATCG			

*fimH*	*fimH*	F: TTATGGCGGCGTGTTATC			
		R: TCCCTACTGCTCCTAACG	545	10	[27]

*Note:* Adapted from Guzmán-Hernández et al. [28].

**Table 2:** Primer sequences and amplicon sizes used in the phylo-typing method.

**Protocol**	**Gene**	**Sequence (5**⁣′**-3**⁣′**)**	**Size (pb)**	**Concentration (pMol)**	**Reference**
Quadruplex	*chuA*	chuA.1b ATGGTACCGGACGAACCAAC	288	0.2	[11]
		chuA.2 GCCGCCAGTACCAAAGACA			
	*yjaA*	yjaA.1b CAAACGTGAAGTGTCAGGAG	211	0.2	
		yjaA.2b AATGCGTTCCTCAACCTGTG			
	TspE4.C2	TspE4C2.1b CACTATTCGTAAGGTCATCC	152	0.2	
		TspE4C2.2b AGTTTATCGCTGCGGGTCGC			
	*arpA*	AceK.f AACGCTATTCGCCAGCTTGC	785	0.2	
		ArpA1.r TCTCCCCATACCGTACGCTA			

Group E	*arpA*	ArpAgpE.f GATTCCATCTTGTCAAAATATGCC	301	0.2	[30]
		ArpAgpE.r GAAAAGAAAAAGAATTCCCAAGAG			
Group C	*trpA*	trpAgpC.1 AGTTTTATGCCCAGTGCGAG	219	0.2	
		trpAgpC.2 TCTGCGCCGGTCACGCCC			

Group G	*ybgD*	ybgD.1 TATGCGGCTGATGAAGGATC-	177	0.2	[12]
		ybgD.2 GTTGACTAAGCGCAGGTCGA			
Group F	*cfaB*	cfaB.1 CTAACGTTGATGCTGCTCTG	384	0.2	
		cfaB.2 TGCTAACTACGCCACGGTAG			

**Table 3:** Primer sequences and amplicon sizes of *β*-lactamase genes.

**Protocol**	**Gene**	**Sequence (5**⁣′**-3**⁣′**)**	**Size (pb)**	**Concentration (pMol)**	**Reference**
*blaOXA48*	*blaOXA48*	F: GATTTTTCGATGGGACGGCG	500 pb	0.4	[32]
		R: ATAGAGCGAAGGATTGCCCG			
*blaCTX-M1-8*	*blaCTX-M1-8*	F: TGTGCAGYACCAGTAARGYKATG	583 pb	0.4	
		R: TARRTSACCAGAAYVAGCGGC			
*blaCTX-M9*	*blaCTX-M9*	F: ATGGTGACAAAGAGAGTGCAA	747 pb	0.4	
		R: AATATCATTGGTGGTGCCGTAG			

**Table 4:** Patient characteristics and origin of the isolates.

**Patients**	**Isolates (*N* = 200)**									
	**Urinary culture** (**n** = 163**)**	**Wound** (**n** = 18**)**	**Sputum** (**n** = 6**)**	**Blood culture** (**n** = 4**)**	**Catheter** (**n** = 3**)**	**Peritoneal fluid** (**n** = 2**)**	**Bronchial aspirate** (**n** = 1**)**	**Ascitic fluid** (**n** = 1**)**	**Otic** (**n** = 1**)**	**Hemodialysis** (**n** = 1**)**
Male	74	11	4	1	2	1	—	1	1	—
Female	89	7	2	3	1	1	1	—	—	1
Age range	9–93 yo	32–84 yo	43–81 yo	41–69 yo	38–62 yo	62–69 yo	78 yo	71 yo	69 yo	48 yo
*Wards*										
Ambulatory surgery	1	—	—	—	—	—	—	—	—	—
General surgery	11	3	1	1	—	—	—	—	—	—
Outpatient consultation	28	2	—	—	—	—	—	—	1	—
Hemodialysis	9	1	1	—	—	—	—	—	—	—
Internal medicine	21	2	2	—	—	—	—	—	—	—
Neurosurgery	3	2	1	1	—	—	—	—	—	—
Pediatrics	1	—	—	—	—	—	—	—	—	—
Operating room	2	—	—	—	—	—	—	—	—	—
Traumatology	5	1	—	—	—	—	—	—	—	—
Intensive care unit	5	—	—	1	1	—	—	—	—	—
Pediatric intensive care unit	1	—	—	1	—	—	—	—	—	—
Adult emergencies	47	2	1	—	1	—	—	1	—	1
Pediatric emergencies	2	5	—	—	1	—	—	—	—	—
Unspecified service	27	—	—	—	—	—	—	—	—	—

**Table 5:** Prevalence of virulence factors in phylogenetic groups of extraintestinal *Escherichia coli* isolates.

**Phylogroup**			**A**	**B1**	**B2**	**C**	**D**	**E**	**F**	**G**	**UNK**
Virulence factors		**n**	27	16	92	8	17	21	9	—	10
		**(%)**	(13.5)	(8)	(46)	(4)	(8.5)	(10.5)	(4.5)	—	(5)

Adhesins	*papC*	124	16	14	63	6	10	6	—	—	7
		(62)	(12.9)	(11.2)	(50.8)	(4.8)	(8.0)	(4.8)	—	—	(5.6)
	*fimA*	68	6	2	35	4	5	9	2	—	5
		(34)	(8.8)	(2.9)	(51.4)	(5.8)	(7.3)	(13.2)	(2.9)	—	(7.3)
	*fimH*	191	26	16	86	9	16	20	8	—	10
		(95.5)	(13.6)	(8.3)	(45.0)	(4.7)	(8.3)	(10.4)	(4.1)	—	(5.2)

Toxins	*vat*	49	2	—	32	1	6	1	1	—	6
		(24.5)	(3.7)	—	(65.3)	(2.0)	(12.2)	(2.0)	(2.0)	—	(12.2)
	*hlyA*	33	2	—	25	—	4	—	—	—	2
		(16.5)	(6.0)	—	(75.7)	—	(12.1)	—	—	—	(6.0)

Nutrition	*iroN*	20	2	2	7	2	4	1	—	—	2
		(10)	(10)	(10)	(35)	(10)	(20)	(5)	—	—	(10)
	*fyuA*	126	15	5	65	5	11	10	5	—	10
		(63)	(11.9)	(3.9)	(51.5)	(3.9)	(8.7)	(7.9)	(3.9)	—	(7.9)
	*iutA*	148	23	11	67	8	12	15	4	—	8
		(74)	(15.5)	(7.4)	(45.2)	(5.4)	(8.1)	(10.1)	(2.7)	—	(5.4)

Membrane	*agn43*	164	17	13	82	6	14	17	6	—	9
		(82)	(10.3)	(7.9)	(50)	(3.6)	(8.5)	(10.3)	(3.6)	—	(5.4)
	*kpsmTII*	69	3	3	45	2	7	4	2	—	3
		(34.5)	(4.3)	(4.3)	(65.2)	(2.8)	(10.1)	(5.7)	(2.8)	—	(4.3)

*Note: N* = 200.

Abbreviation: UNK, unknown.

**Table 6:** Most represented profiles of isolates with multiple virulence genes.

**Virulence gene profiles**	**No. of positive isolates**
*hlyA* + *papC* + *fyuA* + *fimA* + *vat* + *iroN* + *kpsmTII* + *agn43* + *iutA + fimH*	2
*hlyA* + *papC* + *fyuA* + *vat* + *iroN* + *kpsmTII* + *agn43* + *iutA + fimH*	2
*hlyA* + *papC* + *fyuA* + *fimA* + *vat* + *iroN* + *agn43* + *iutA + fimH*	2
*agn43* + *iutA + fimH*	8
*hlyA* + *papC* + *fyuA* + *fimA* + *vat* + *kpsmTII* + *agn43* + *iutA + fimH*	9
*papC* + *agn43* + *iutA + fimH*	9
*papC* + *fyuA* + *kpsmTII* + *agn43* + *iutA + fimH*	17

## Data Availability

The data that support the findings of this study are available on request from the corresponding author. The data are not publicly available due to privacy or ethical restrictions.

## Supporting Information

Supporting Information Additional supporting information can be found online in the Supporting Information section. Table S1: Minimum inhibitory concentration (MIC) breakpoints for antimicrobial agents used in susceptibility testing.
