# Supplementary material for: Virulence, Phylogenetic Grouping, and Antimicrobial Resistance Traits of Extraintestinal Escherichia coli in Clinical Isolates From Northwest Mexico
Source: Int J Microbiol. 2025 Sep 19;2025:8881117. doi: 10.1155/ijm/8881117 (PMC12473729; doi:10.1155/ijm/8881117)
Supplement: Supporting Information — Additional supporting information can be found online in the Supporting Information section. Table S1: Minimum inhibitory concentration (MIC) breakpoints for antimicrobial agents used in susceptibility testing. [file 8881117.f1.docx]

Supplementary Table 1. Minimum Inhibitory Concentration (MIC) Breakpoints for Antimicrobial Agents Used in Susceptibility Testing

| **Antimicrobial agent** | **MIC Breakpoint (µg/mL)** |
| --- | --- |
| Ampicillin | >=32 |
| Ampicillin-sulbactam | >=32 |
| Piperacillin-tazobactam | 16 |
| Cefazolin | >=64 |
| Cephalothin | >=64 |
| Cefuroxime | >=64 |
| Cefuroxime-axetil | >=64 |
| Cefotaxime | >=64 |
| Ceftazidime | 16 |
| Ceftriaxone | >=64 |
| Cefepime | 8 |
| Aztreonam | 16 |
| Ertapenem | <=0.5 |
| Meropenem | <=0.25 |
| Amikacin | <=2 |
| Gentamicin | >=16 |
| Tobramycin | >=16 |
| Ciprofloxacin | >=4 |
| Norfloxacin | >=16 |
| Fosfomycin | >=16 |
| Tigecycline | <=0.5 |
| Nitrofurantoin | <=16 |
| Trimethoprim-sulfamethoxazole | >=320 |
| Amoxicillin |  |
